# Saroglitazar Ameliorates Pulmonary Fibrosis Progression in Mice by Suppressing NF-κB Activation and Attenuating Macrophage M1 Polarization

**DOI:** 10.3390/medicina61071157

**Published:** 2025-06-26

**Authors:** Yawen Zhang, Jiaquan Lin, Xiaodong Han, Xiang Chen

**Affiliations:** 1 State Key Laboratory of Analytical Chemistry for Life Science, Division of Anatomy and Histo-Embryology, Medical School, Nanjing University, Nanjing 210093, China; zhangyawen302@163.com; 2 Jiangsu Key Laboratory of Molecular Medicine, Nanjing University, Nanjing 210093, China; jemery_q1021@163.com; 3 School of Medicine, Nanjing University, Nanjing 210093, China

**Keywords:** pulmonary fibrosis, inflammation, saroglitazar, M1 macrophage polarization, NF-κB pathway

## Abstract

*Background and Objectives*: Idiopathic pulmonary fibrosis (IPF) is a progressive and fatal interstitial lung disease with limited therapeutic options. Current therapies (pirfenidone, nintedanib) exhibit modest efficacy and significant side effects, underscoring the need for novel strategies targeting early pathogenic drivers. Saroglitazar (SGZ), a dual PPARα/γ agonist with anti-inflammatory properties approved for diabetic dyslipidemia, has not been explored for IPF. We aimed to investigate SGZ’s therapeutic potential in pulmonary fibrosis and elucidate its mechanisms of action. *Materials and Methods*: Using a bleomycin (BLM)-induced murine pulmonary fibrosis model, we administered SGZ therapeutically. A histopathological assessment (H&E, Masson’s trichrome, collagen I immunofluorescence), Western blotting, and qRT-PCR analyzed the fibrosis progression and inflammatory markers. Flow cytometry evaluated the macrophage polarization. In vitro studies used RAW264.7 macrophages stimulated with BLM/LPS and MRC-5 fibroblast co-cultures. The NF-κB/NLRP3 pathway activation was assessed through protein and gene expression. *Results*: SGZ significantly attenuated BLM-induced histopathological hallmarks, including alveolar wall thickening, collagen deposition, and inflammatory infiltration. Fibrotic markers (OPN, α-SMA) and pro-inflammatory cytokines (IL-1β, TNF-α, IL-6) were downregulated in the SGZ-treated mice. Mechanistically, SGZ suppressed the M1 macrophage polarization (reduced CD86^+^ populations) and inhibited the NF-κB/NLRP3 pathway activation in the alveolar macrophages. In the RAW264.7 cells, SGZ decreased the NLRP3 inflammasome components (ASC, cleaved IL-1β) and cytokine secretion. Co-cultures demonstrated that the SGZ-treated macrophage supernatants suppressed the fibroblast activation (α-SMA, collagen I) in MRC-5 cells. *Conclusions*: SGZ attenuates pulmonary fibrosis by suppressing macrophage-driven inflammation via NF-κB/NLRP3 inhibition and disrupting the macrophage–fibroblast crosstalk. These findings nominate SGZ as a promising candidate for preclinical optimization and future clinical evaluation in IPF.

## 1. Introduction

Idiopathic pulmonary fibrosis is a progressive and fatal interstitial lung disease characterized by excessive collagen deposition, alveolar destruction, and irreversible respiratory failure [1,2,3]. Current therapies, including pirfenidone and nintedanib, only modestly slow the disease progression and are associated with significant side effects such as hepatotoxicity and gastrointestinal complications [4,5,6,7,8]. The lack of treatments targeting early pathogenic drivers underscores the urgent need for novel therapeutic strategies focused on immune modulation.

Macrophages play a pivotal role in IPF pathogenesis by dynamically shifting between pro-inflammatory M1 and anti-inflammatory M2 phenotypes [9]. While acute M1 activation is essential for host defense, sustained M1 polarization exacerbates tissue injury by promoting chronic inflammation and epithelial cell apoptosis [10]. In early IPF stages, M1 macrophages dominate, releasing cytokines such as TNF-α, IL-6, and reactive oxygen species that exacerbate epithelial injury and fibroblast activation [11,12]. Clinical studies have shown elevated M1 markers CD86 and iNOS in the bronchoalveolar lavage fluid of IPF patients, correlating with disease severity [13,14,15]. As the disease progresses, the excessive activation of inflammation levels can exacerbate the necrosis of lung tissue [16,17]. For instance, elevated M2-derived TGF-β1 shifted by the M1 macrophage has been linked to fibroblast-to-myofibroblast transition, while ROS overproduction directly damages alveolar epithelial cells, further perpetuating fibrotic remodeling [9,18,19,20]. Thus, reprogramming macrophage polarization represents a promising therapeutic avenue. Nuclear factor-kappa B (NF-κB) is a master regulator of M1 macrophage activation [21,22,23,24,25]. Upon stimuli such as bleomycin or TGF-β, NF-κB translocates to the nucleus, driving the transcription of pro-inflammatory genes such as COX-2 and CCL2 [18,19]. Preclinical studies demonstrate that NF-κB inhibition reduces lung fibrosis in mice; however, existing inhibitors (e.g., ACT001) face clinical limitations due to systemic toxicity [26]. Recent evidence suggests that peroxisome proliferator-activated receptor gamma (PPARγ) agonists may indirectly suppress NF-κB through transrepression mechanisms [27,28,29]. For instance, in diabetic models, PPARγ activation attenuated renal inflammation by blocking the NF-κB nuclear translocation in macrophages [30,31].

Saroglitazar, a dual PPARα/γ agonist approved for diabetic dyslipidemia, exhibits potent anti-inflammatory properties [32,33]. In a murine non-alcoholic steatohepatitis (NASH) model, saroglitazar reduced the hepatic M1 macrophage infiltration by 40% and suppressed the NF-κB activity, highlighting its immunomodulatory potential beyond metabolic regulation [34,35,36,37]. Despite these findings, its role in IPF—particularly in modulating lung macrophage polarization—remains unexplored.

Here, we hypothesized that saroglitazar mitigates IPF by inhibiting the NF-κB-mediated M1 macrophage polarization. Using a bleomycin-induced murine fibrosis model, we demonstrated that saroglitazar significantly reduced collagen deposition and improved lung function. Mechanistically, it suppressed the NF-κB activation in alveolar macrophages, inhibiting their M1 phenotype. Our study identifies saroglitazar as a dual-targeting agent against IPF, leveraging both metabolic and anti-inflammatory pathways, with high translational potential given its established clinical safety.

## 2. Methods and Materials

### 2.1. Animal

Male C57BL/6J mice were obtained from Zhejiang Ziyuan Laboratory Animal Co., Ltd. (Hangzhou, China). The animals were individually housed in ventilated cages within a barrier-maintained SPF facility, where the environmental conditions, including temperature (22 ± 1 °C) and humidity (55 ± 5%), were strictly controlled. A standardized photoperiod of 12 h light and 12 h darkness was maintained throughout the study, with ad libitum access to autoclaved rodent feed and sterile water. The Institutional Animal Ethics Committee of Nanjing University approved all experimental protocols.

### 2.2. Bleomycin-Induced Pulmonary Fibrosis Model and Saroglitazar’s Treatment

Eight-week-old male C57BL/6J mice were randomized into three groups (*n* = 10/group): (1) saline (control), (2) bleomycin-induced pulmonary fibrosis model (BLM), (3) BLM + saroglitazar-treated BLM mice (BLM + SGZ). Pulmonary fibrosis was induced by a single intratracheal instillation of bleomycin (3.0 U/kg, MCE HY-17565, Shanghai, China) under 2% isoflurane anesthesia. Starting at 24 h post-BLM administration, the saroglitazar (4 mg/kg/day, MCE HY-19937, Shanghai, China) or vehicle (saline) was administered orally for 21 consecutive days. On day 22, the mice were euthanized for tissue collection for further experiments.

### 2.3. Cell Culture and Drug Administration

The RAW264.7 cells (Pricella, CL-0190, Wuhan, China) and the MRC-5 cells (Pricella, CL-0161, Wuhan, China) were cultured in DMEM (Gibco, 11965092, San Francisco, CA, USA) containing 10% FBS (Gibco, A5670701, San Francisco, CA, USA) and 1% penicillin/streptomycin (Gibco, 15140122) at 37 °C with 5% CO_2_, passaged at 80% confluency. For the inflammatory modeling, the cells were seeded in 6-well plates (2 × 10^5^ per well) and stimulated with 50 mU/mL bleomycin for 24 h or 100 ng/mL lipopolysaccharide (LPS, Solarbio, L8880, Beijing, China) for 6 h. To evaluate saroglitazar’s anti-inflammatory effects, the cells were pre-treated with 10 μM saroglitazar for 2 h before the BLM/LPS exposure or co-treated for the indicated durations.

### 2.4. Hematoxylin and Eosin (H&E) Staining

The lung tissues were fixed in 4% paraformaldehyde for 48 h, dehydrated through a graded ethanol series, and embedded in paraffin. The sections (4 μm thickness) were deparaffinized with xylene, rehydrated in descending ethanol concentrations, and stained with Harris hematoxylin (Solarbio, G1120, Beijing, China) for 5 min. After differentiation in 1% acid ethanol and bluing in warm tap water (pH 8.5), the sections were counterstained with eosin for 1 min. Following the dehydration and clearing, the slides were mounted with neutral balsam and imaged under a light microscope magnification.

### 2.5. Masson’s Trichrome Staining

The paraffin-embedded lung sections (4 μm) were dewaxed in xylene, rehydrated through graded ethanol, and stained using a Masson’s trichrome kit (Solarbio, G1346, Beijing, China) according to the manufacturer’s protocol. Briefly, the nuclei were stained with Weigert’s iron hematoxylin for 10 min, followed by differentiation in 1% acid ethanol. The sections were then immersed in the Biebrich scarlet-acid fuchsin solution for 5 min, treated with phosphomolybdic-phosphotungstic acid for 10 min, and counterstained with aniline blue for 5 min to visualize the collagen fibers. The slides were imaged under a light microscope after dehydration and mounting with neutral resin.

### 2.6. Ashcroft Scoring Method

The severity of pulmonary fibrosis was quantified using the semi-quantitative Ashcroft scoring system, as previously validated. Briefly, the hematoxylin and eosin (H&E)-stained lung sections (5-μm thickness) were systematically evaluated under light microscopy at 100× magnification. The entire sections were divided into a grid of non-overlapping fields (≥5 fields per lung). Two independent pathologists, blinded to the experimental groups, assigned each field an integer score from 0 to 8 based on predefined architectural alterations. The final Ashcroft scores represent the average of both pathologists’ means.

### 2.7. Immunofluorescence Staining

The fresh lung tissues were embedded in an OCT compound (Sakura, SA62550-01, Tokyo, Japan), snap-frozen in liquid nitrogen, and cryosectioned at 8 μm thickness. The sections were fixed in 4% PFA (10 min, RT), permeabilized with 0.3% Triton X-100 (15 min), and blocked with 5% donkey serum (Yeasen, 36136ES60, Nanjing, China) for 1 h. The primary antibody against collagen I (1:1000, Servicebio GB115707-100, Wuhan, China) was applied overnight at 4 °C, followed by Alexa Fluor 488-conjugated secondary antibody (1:1000, Invitrogen A-11008, Carlsbad, CA, USA) for 2 h. The nuclei were stained with DAPI (5 min). The images were acquired using a confocal microscope (Olympus FV3000, Tokyo, Japan), and the fluorescence intensity was quantified in five random fields/section (400×) using Image J v1.54. All of the fluorescence intensity quantifications were specifically normalized to the number of nuclei.

### 2.8. Western Blot Analysis

The lung tissues were homogenized in RIPA lysis buffer (Beyotime, P0013B, Beijing, China) containing protease/phosphatase inhibitors (Roche, 4906837001, Basel, Switzerland). The protein concentrations were determined via BCA assay (Beyotime, P0012, Beijing, China). The samples (30 μg/lane) were separated on 10% SDS-PAGE gels and transferred to PVDF membranes (Millipore, IPVH00010, Hub Carlsbad, CA, USA). After blocking with 5% non-fat milk for 1 h, the membranes were incubated overnight at 4 °C with primary antibodies: collagen I (1:1000, Servicebio GB115707-100, Wuhan, China), OPN (1:1000, Servicebio GB112328-100, Wuhan, China), α-SMA (1:1000, Servicebio GB111364-100, Wuhan, China), IL-6 (1:1000, Proteintech 21865-1-AP, Wuhan, China), IL-1β (1:1000, CST D3U3E, Waltham, MA, USA), TNF-α (1:1000, ABclonal A22227, Nanjing, China), NLRP3 (1:5500, ABclonal A24294, Nanjing, China), ASC (1:1000, ABclonal A22046, Nanjing, China), and p-NF-κB p65 (1:500, ABclonal AP0125, Nanjing, China), with GAPDH (1:1000, Proteintech 10494-1-AP, Wuhan, China) and β-actin (1:1000, Proteintech 20536-1-AP, Wuhan, China) as the loading controls. HRP-conjugated secondary antibodies (1:5000, Proteintech SA00001-2/SA00001-1, Wuhan, China) were applied for 1 h at RT. Signals were detected using the ECL reagent (Yeasen, 36223ES76, Nanjing, China) and quantified by Image Lab 6.1 (Bio-Rad, Hercules, CA, USA).

### 2.9. RNA Isolation and Quantitative Real-Time PCR

The total RNA was isolated from the tissues or cultured cells using the RNAiso™ Plus reagent (Takara Bio Inc., 9109, Tokyo, Japan) following the manufacturer’s instructions. Briefly, the homogenized samples were incubated with 1 mL RNAiso™ Plus at 25 °C for 5 min, followed by the addition of 200 μL chloroform and vigorous vortexing for 30 s. After centrifugation (12,000× *g*, 4 °C, 10 min), the aqueous phase was transferred to fresh tubes and mixed with 500 μL isopropanol. RNA pellets were obtained by centrifugation (12,000× *g*, 4 °C, 10 min), washed twice with 75% ethanol, and dissolved in 30 μL RNase-free water (Servicebio, G4700, Wuhan, China). The RNA purity and concentration were assessed spectrophotometrically. Next, a first-strand cDNA synthesis was performed using PrimeScript™ RT Master Mix (Takara Bio, RR036B, Tokyo, Japan) with 1 μg total RNA. Quantitative PCR was conducted on a QuantStudio 6 Flex system (Applied Biosystems, Foster City, CA, USA) using TB Green^®^ Premix Ex Taq™ II (Takara Bio, RR82WR, Tokyo, Japan). Reaction conditions: 95 °C for 30 s, 40 cycles of 95 °C (5 s), and 60 °C (30 s). The target gene expression (collagen I, α-SMA, IL-6, IL-1β, TNF-α, NLRP3, ASC) was normalized to β-actin and GAPDH using the 2^−ΔΔCt^ method. The primer sequences are provided in Supplementary Table S1.

### 2.10. Enzyme-Linked Immunosorbent Assay (ELISA)

The fresh lung tissues were homogenized in ice-cold PBS supplemented with protease inhibitors using a Precellys 24 homogenizer (Bertin Instruments, 3 × 30 s cycles at 6500 rpm). The homogenates were centrifuged (12,000× *g*, 20 min, 4 °C), and the supernatants were collected for analysis. The levels of IL-1β, TNF-α, and IL-6 were quantified using species-specific ELISA kits (IL-6: Abcam, ab222503, Cambridge, UK; TNF-α: Abcam, ab208348, Cambridge, UK; IL-1β: Abcam, ab197742, Cambridge, UK) following the manufacturers’ protocols. Briefly, 50 μL of the supernatant or standards were loaded onto antibody-precoated plates and incubated (2 h, 25 °C). After washing (0.1% Tween-20/PBS), biotinylated detection antibodies were added (1 h, 25 °C), followed by streptavidin-HRP (30 min) and TMB substrate (15 min, protected from light), and the absorbance was measured at 450/630 nm using a SpectraMax i3x plate reader (Molecular Devices, San Jose, CA, USA). The tissue cytokine concentrations were normalized to the total protein content (μg/mg tissue), as determined by a BCA assay kit (Beyotime, P0012, Beijing, China).

### 2.11. Flow Cytometry Analysis

The lung single-cell suspensions were prepared by digesting tissues with collagenase IV (1 mg/mL, Gibco, P5755103) and DNase I (50 U/mL, Sigma, 18047019, San Jose, CA, USA) for 45 min at 37 °C, followed by filtration through 70-μm cell strainers. After RBC lysis (BD Biosciences, 555899, Franklin Lakes, NJ, USA), the cells were stained with FITC-anti-CD45 (BD Biosciences, 561088, Franklin Lakes, NJ, USA), PE-CY7-anti-CD86 (BD Biosciences, 560582, New Jersey, USA), and APC-anti-CD11b (BD Biosciences, 567499, Franklin Lakes, NJ, USA) for 30 min at 4 °C in PBS containing 2% FBS. Fluorescence minus one (FMO) controls were used for gating optimization. Flow cytometry was performed on a BD FACSAria III (BD Biosciences, Franklin Lakes, NJ, USA), and the M1 macrophages were defined as CD45^+^ CD11b^+^ CD86^+^ populations: briefly, using 7-AAD to remove the dead cell from the single-cell population; next, staining with CD45 and CD11b to obtain the macrophage population and applying the CD86 to obtain the M1 macrophages population. The data were analyzed using FlowJo10.8, with the results expressed as the percentage of CD86^+^ cells within the total macrophages.

### 2.12. Statistical Analysis

All of the quantitative data were expressed as mean ± standard error of the mean (SEM) from at least three independent experiments. The statistical analyses were performed using GraphPad Prism 9.0. For the multiple group comparisons, one-way analysis of variance (ANOVA) followed by Tukey’s post hoc test was applied; *p* < 0.05 was considered statistically significant.

## 3. Results

### 3.1. Saroglitazar Ameliorates the Progression of IPF

**Figure 1:**
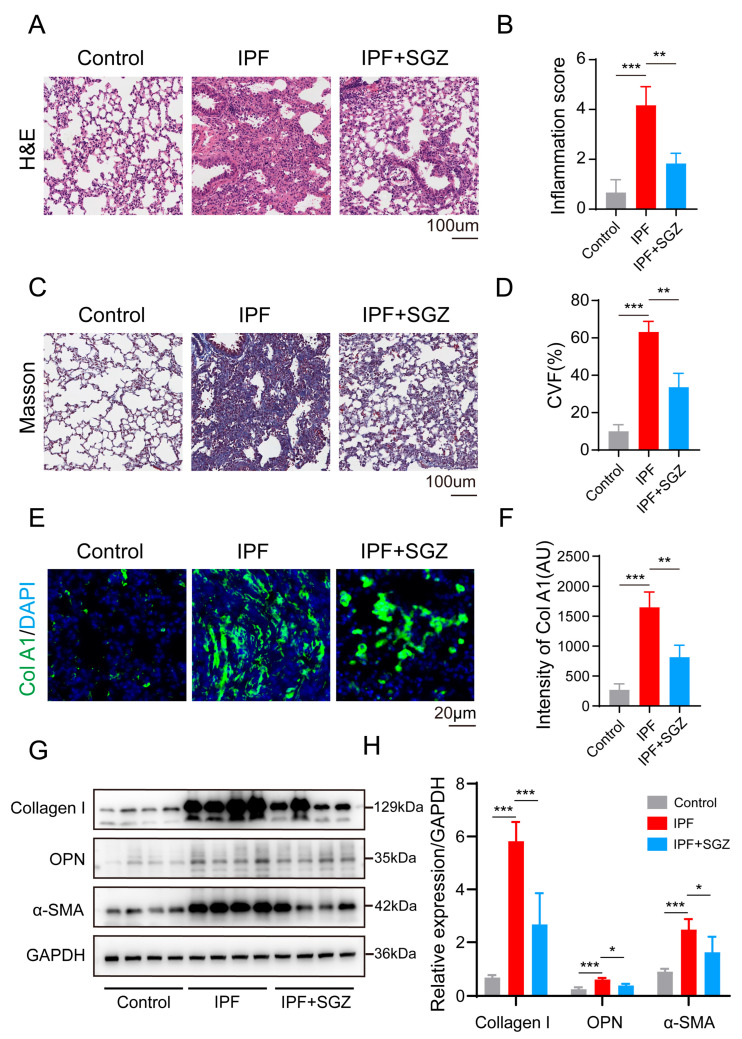
Saroglitazar ameliorates progression of BLM. (**A**) HE staining of lung tissue in control, BLM, and BLM mice treated with SGZ, scale bar = 100 μm. N = 3. (**B**) Statistical analysis of HE staining in A. (**C**) Masson staining of lung tissue in control, BLM, and BLM mice treated with SGZ, scale bar = 100 μm. N = 3. (**D**) Statistical analysis of Masson staining in C. (**E**) IF staining of collagen 1 of lung tissue in control, BLM, and BLM mice treated with SGZ, scale bar = 20 μm. N = 3. (**F**) Statistical analysis of IF staining in E. (**G**) Western blot analysis of lung tissue of control, BLM, and BLM mice treated with SGZ. N = 4. (**H**) Statistical analysis of WB in G. * means *p* < 0.05; ** means *p* < 0.01; *** means *p* < 0.001.

To reveal the effect of saroglitazar (SGZ) on the progression of pulmonary fibrosis in mice, we successfully established an IPF model using a single intratracheal instillation of bleomycin. Subsequently, saroglitazar was administered orally to the BLM mice to enhance its therapeutic benefits. We employed HE staining to evaluate the role of saroglitazar from a histopathological standpoint (Figure 1A). The analysis of the results indicated reduced alveolar wall thickening, inflammatory cell infiltration, and structural distortion in the saroglitazar-treated mice (BLM + SGZ) compared to the BLM group. The inflammation score decreased following the saroglitazar administration (Figure 1B), suggesting that the application of SGZ altered the trajectory of the IPF disease development. To confirm these findings, we performed Masson staining next (Figure 1C). We assessed the collagen deposition in the lung tissue of each group of mice, and the results showed that the percentage of collagen volume fraction (CVF) increased after the intratracheal instillation of bleomycin compared to the control group, indicating the successful establishment of the BLM model. Compared with the BLM group, the percentage of CVF in the BLM + SGZ group decreased, which is consistent with the HE staining results (Figure 1D). We also evaluated the collagen deposition using immunofluorescence staining of collagen I (Figure 1E). Consistent with the Masson staining results, the treatment with SGZ significantly reduced the expression level of collagen (Figure 1F). Next, we evaluated the effects from the perspective of protein molecules. We detected the expression levels of pulmonary fibrosis markers OPN and α-SMA through a Western blot (WB) analysis of the lung tissue (Figure 1G). The treatment with SGZ significantly reduced the levels of OPN and SMA in the lung tissue of mice 21 days after modeling (Figure 1H). All of these results suggest that SGZ treatment can significantly improve the tissue damage and inflammatory infiltration in BLM mice lung tissue, inhibit the collagen fiber deposition in lung tissue, and delay the progression of fibrosis.

### 3.2. Saroglitazar Attenuates M1 Polarization of Macrophages in BLM Mice

**Figure 2:**
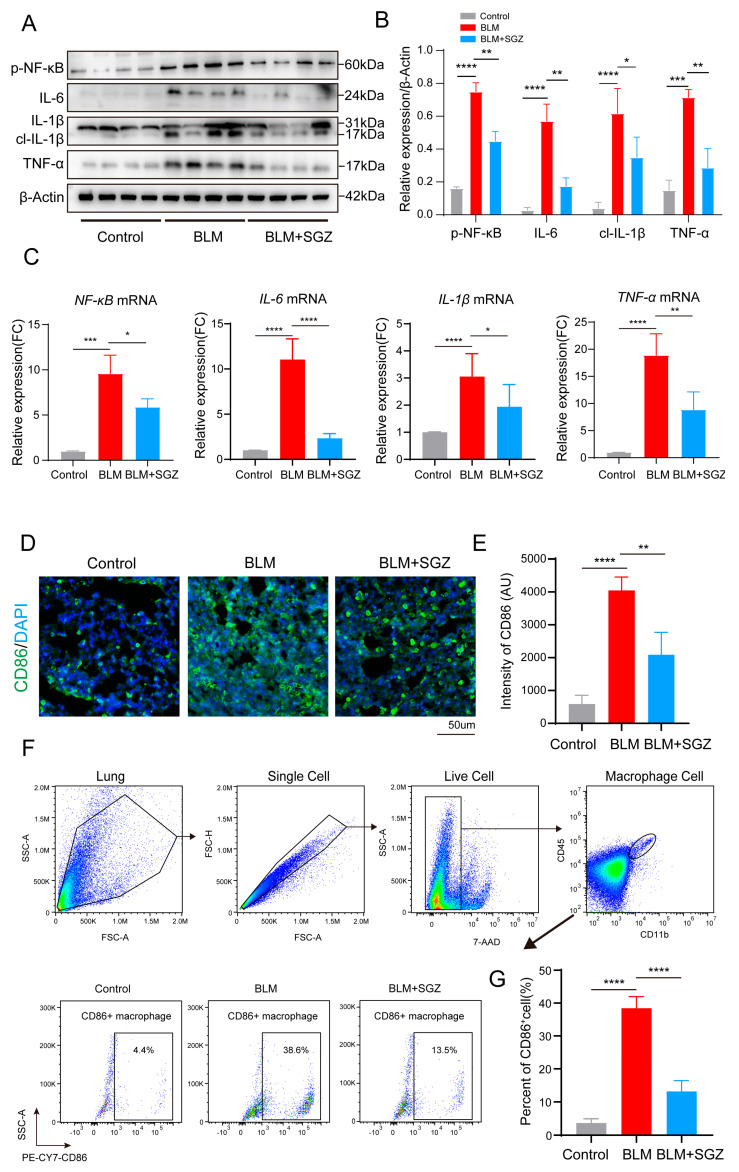
Saroglitazar attenuates M1 polarization of macrophages in BLM mice. (**A**) Western blot analysis of lung tissue of control, BLM, and BLM mice treated with SGZ. N = 4. (**B**) Statistical analysis of WB in A. (**C**) PCR analysis of IL-1β, IL-6, and TNF-α of lung tissue in control, BLM, and BLM mice treated with SGZ. N = 3. (**D**) IF staining of CD86 in lung tissue of control, BLM, and BLM mice treated with SGZ, scale bar = 50 μm. N = 3. (**E**) Statistical analysis of IF staining in (**D**). (**F**) FCM analysis of CD86-positive macrophage cells in lung tissue of control, BLM, and BLM mice treated with SGZ. N = 6. (**G**) Statistical analysis of FCM in (**F**). * means *p* < 0.05; ** means *p* < 0.01; *** means *p* < 0.001 and **** means *p* < 0.0001.

To verify the observation of inflammatory infiltration in Figure 1A, we aimed to understand the specific regulatory effect of SGZ on the inflammatory response. We evaluated the inflammatory levels of lung tissue in each group of mice through Western blot analysis (Figure 2A). We found that the expression levels of p-NF-κB, IL1, IL6, and TNF-α were significantly upregulated in the pulmonary fibrosis model mice, while their expression was significantly inhibited after treatment with SGZ (Figure 2B). Simultaneously, we observed the same trend of changes at the transcriptional level through PCR analysis (Figure 2C). As is known, macrophages play a crucial role in the inflammation reaction of mouse pulmonary fibrosis. We then evaluated the CD86 (M1 macrophage marker) expression in the lung tissue after SGZ treatment by IF staining (Figure 2D), and the results showed that the IF intensity of the CD86 protein was decreased in the BLM + SGZ group compared with the BLM group (Figure 2F). Next, we focused on the polarization of M1 macrophages. We prepared a single-cell suspension from mouse lung tissue homogenate and analyzed the M1 polarized macrophage subpopulation using flow cytometry. We extracted CD45^+^ CD11b^+^ macrophages and further analyzed them, finding that the treatment with SGZ significantly decreased the percentage of CD86 cell subsets in the BLM mice (Figure 2G), which is consistent with the results of the CD86 IF staining. All of these results indicated that saroglitazar attenuated the M1 polarization of macrophages and inhibited the inflammation in BLM mice.

### 3.3. Saroglitazar Suppresses the Activation of Inflammation in RAW264.7 Induced by Bleomycin

**Figure 3:**
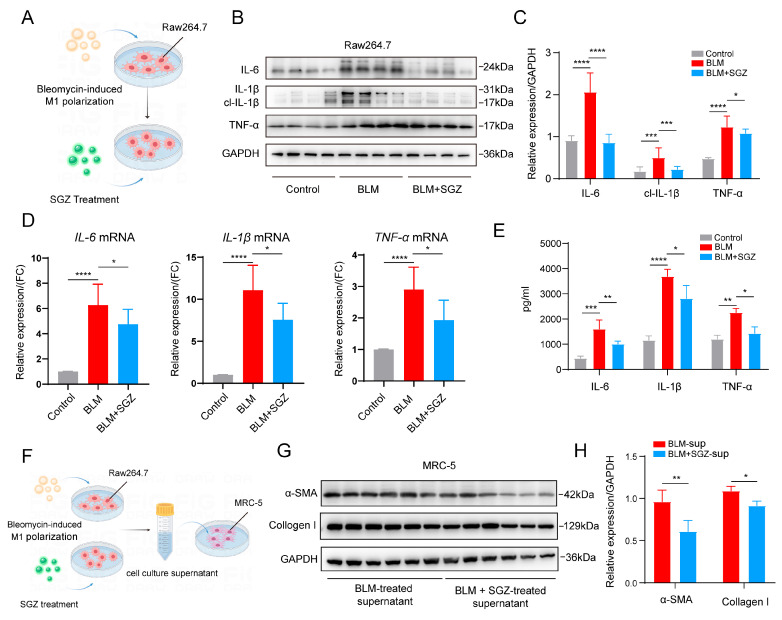
Saroglitazar suppresses the activation of inflammation of RAW264.7 induced by bleomycin. (**A**) Schematic diagram of BLM and SGZ treatment with RAW264.7. (**B**) Western blot analysis of RAW264.7 treated with BLM and SGZ. N = 4. (**C**) Statistical analysis of WB in (**B**). (**D**) PCR analysis of IL-1β, IL-6, and TNF-α in RAW264.7 treated with BLM and SGZ. N = 4. (**E**) ELISA analysis of IL-1β, IL-6, and TNF-α of RAW264.7 treated with BLM and SGZ. N = 3. (**F**) Schematic diagram of co-culture system. (**G**) Western blot analysis of MRC-5 cells treated with supernatant. N = 6. (**H**) Statistical analysis of WB in (**G**). * means *p* < 0.05; ** means *p* < 0.01; *** means *p* < 0.001 and **** means *p* < 0.0001.

To validate the anti-inflammatory effect of SGZ in vivo, we constructed a pulmonary fibrosis macrophage activation model by treating RAW264.7 cells with BLM in vitro (Figure 3A). We found that the administration of BLM significantly enhanced the protein expression of IL-6, IL-1β, and TNF-α in the lung tissue, and treatment with SGZ remarkably decreased these proteins (Figure 3B,C). Simultaneously, we observed the same trend of changes at the transcriptional level through PCR analysis (Figure 3D), with the mRNA levels of *IL-6*, *IL-1β*, and *TNF-α* downregulated after the SGZ treatment. On this basis, we collected cell supernatants treated with BLM and SGZ, and through ELISA analysis, we found that the secretion level of the inflammatory cytokines IL-6, IL-1β, and TNF-α also significantly decreased (Figure 3E). All of these results are consistent with the experimental data in vivo, represented in Figure 2.

Next, we investigated the impact of SGZ-induced immune inflammation on pulmonary fibrosis through co-culture systems. We collected the culture supernatants of RAW264.7 cells treated with BLM and BLM + SGZ, respectively, and added them to the MRC-5 cells (Figure 3F). We evaluated the α-SMA and collagen 1 protein levels, and the results showed that the BLM-treated supernatants increased the protein levels of α-SMA and collagen 1, while the SGZ treatment reversed those changes (Figure 3G,H). All of these results demonstrated that saroglitazar could suppress the activation of inflammation in RAW264.7 induced by bleomycin.

### 3.4. Anti-Inflammation of Saroglitazar Depends on the NF-κB/NLRP3 Pathway

**Figure 4:**
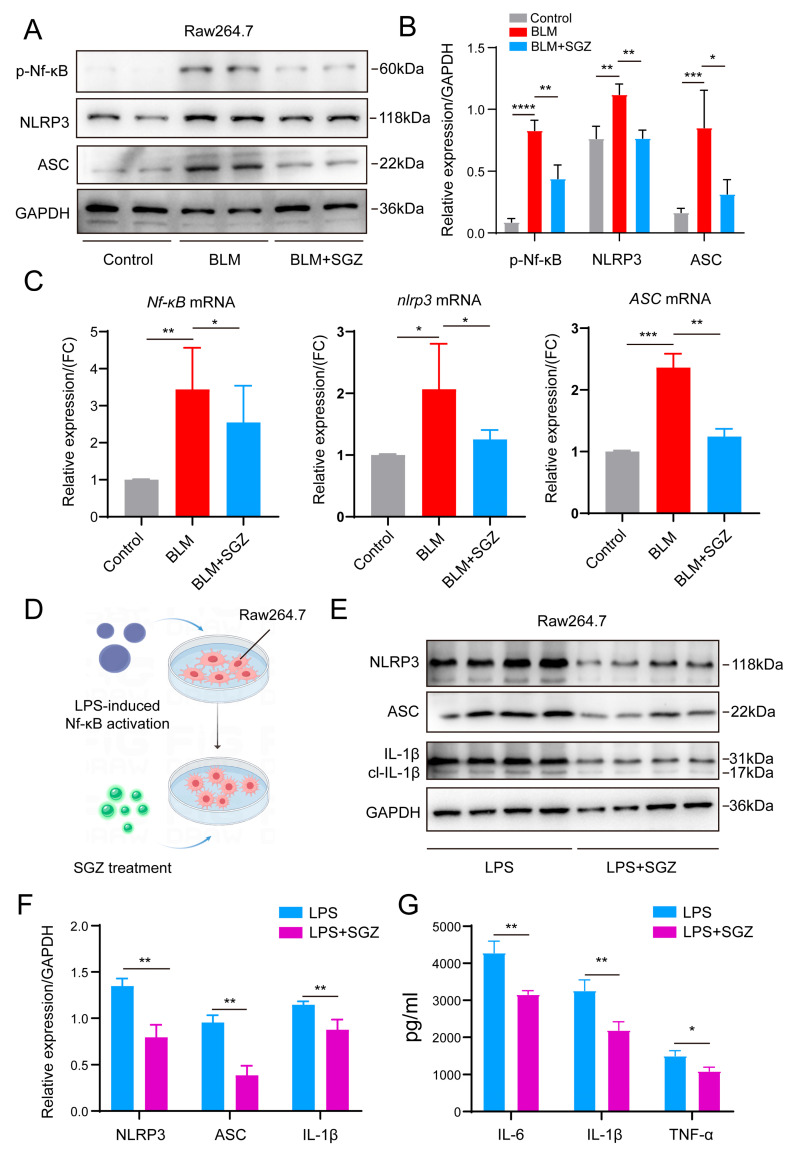
Anti-inflammation of saroglitazar depends on NF-κB/NLRP3 pathway. (**A**) Western blot analysis of RAW264.7 treated with BLM and SGZ. N = 4. (**B**) Statistical analysis of WB in A. (**C**) PCR analysis of RAW264.7 treated with BLM. N = 4. (**D**) Schematic diagram of LPS and SGZ treatment. (**E**) Western blot analysis of RAW264.7 treated with LPS and SGZ. N = 4. (**F**) Statistical analysis of WB in (**E**). (**G**) ELISA analysis of IL-1β, IL-6, and TNF-α in RAW264.7 treated with LPS and SGZ. N = 3. * means *p* < 0.05; ** means *p* < 0.01; *** means *p* < 0.001 and **** means *p* < 0.0001.

Having observed the negative regulatory effect of SGZ on the M1 polarization of macrophages, we sought to understand the potential molecular mechanisms that have yet to be revealed. It is understood that NF-κB is crucial to the activation of macrophages, and its downstream NLRP3 and ASC proteins control the polarization of M1 macrophages. We applied a WB analysis to detect the NF-κB/NLRP3 pathway of RAW264.7 treated with BLM and SGZ, as illustrated in Figure 3A. The results showed that the BLM-induced activation of RAW264.7 significantly upregulated the p-NF-κB, NLRP3, and ASC proteins, while the treatment of SGZ could downregulate the protein expression (Figure 4A,B). Simultaneously, we observed the same trend of changes at the transcriptional level through PCR analysis (Figure 4C).

Next, we administered LPS (the classical NF-κB activator) instead of the BLM treatment. We applied a WB analysis to detect the NLRP3/ASC/IL-1β signal pathway (Figure 4D). Interestingly, we found that the SGZ administration also significantly decreased the protein expression of the NLRP3/ASC/IL-1β signal pathway compared with the LPS-treated group (Figure 4E,F). We also performed ELISA detection and analysis on the cell culture supernatant of the LPS group and the LPS + SGZ group. The results showed that the secretion level of inflammatory cytokines IL-6, IL-1β, and TNF-α also significantly decreased after treatment with SGZ in RAW264.7 (Figure 4G). Thus, all of these results indicated that saroglitazar inhibits the inflammation induced by BLM and LPS by targeting the NF-κB/NLRP3 pathway.

## 4. Discussion

Saroglitazar (SGZ), a dual PPARα/γ agonist, demonstrates potent therapeutic efficacy in mitigating inflammation through the multifaceted modulation of macrophage-driven polarization [32,33]. Our findings reveal that SGZ treatment significantly attenuates histopathological hallmarks of IPF, including alveolar wall thickening, inflammatory infiltration, and collagen deposition, as evidenced by multimodal analyses (H&E, Masson’s trichrome, and collagen I immunofluorescence). The downregulation of fibrotic markers (OPN, α-SMA) and pro-inflammatory cytokines (IL-1β, TNF-α, IL-6) in SGZ-treated mice underscores its dual antifibrotic and immunomodulatory properties. Crucially, SGZ suppresses M1 macrophage polarization—a pivotal driver of early fibrogenesis—by reducing CD86^+^ M1 macrophage populations in vivo and blunting the NF-κB/NLRP3 pathway activation in vitro. This mechanistic convergence was further validated in LPS-stimulated models, where SGZ inhibited the NLRP3 inflammasome assembly and IL-1β maturation, suggesting broad applicability across diverse inflammatory triggers.

The anti-inflammatory effects of SGZ extend beyond macrophage reprogramming to paracrine regulation of fibroblast activation. Co-culture experiments demonstrated that SGZ-treated macrophage supernatants suppress the α-SMA and collagen I expression in MRC-5 fibroblasts, implicating cytokine-mediated crosstalk as a therapeutic axis. While PPARγ agonists have shown limited clinical success in IPF [38], SGZ’s dual PPARα/γ agonism may synergistically enhance lipid metabolic regulation and inflammasome suppression, offering a strategic advantage over single-target agents.

Despite these advances, several limitations warrant consideration. First, the bleomycin model predominantly reflects acute inflammatory phases of fibrosis, whereas human IPF involves chronic, aging-associated mechanisms that were not recapitulated here. Second, while SGZ robustly inhibited the M1 polarization, its impact on pro-repair M2 macrophages remains unexplored, leaving unresolved whether fibrosis resolution involves macrophage phenotype switching. Third, the PPARγ dependency of SGZ’s NF-κB/NLRP3 inhibition remains unconfirmed; the genetic or pharmacological disruption of PPARγ signaling could clarify the receptor-specific versus off-target effects. Finally, the single-dose regimen (4 mg/kg) and short-term evaluation (21 days) preclude assessment of dose–response relationships or long-term safety—critical parameters for clinical translation. Future studies should integrate single-cell transcriptomics to map SGZ’s impact on lung cellular ecosystems and employ patient-derived organoids to validate its efficacy in human-relevant microenvironments. Elucidating the upstream regulators of NF-κB (e.g., IKKβ ubiquitination) through phosphor-proteomics could refine SGZ’s molecular targeting strategy.

In conclusion, SGZ represents a promising therapeutic candidate for IPF, uniquely bridging metabolic and immune modulation. Via inhibiting the NF-κB/NLRP3-M1 macrophage axis and disrupting the fibroblast activation, it addresses both inflammatory initiation and fibrotic progression. These findings position SGZ for further preclinical optimization and highlight macrophage reprogramming as a viable strategy for combating refractory fibrotic disorders.

## 5. Conclusions

In summary, saroglitazar attenuates pulmonary fibrosis by suppressing macrophage-driven inflammation via NF-κB/NLRP3 inhibition and disrupting the macrophage–fibroblast crosstalk. These findings nominate SGZ as a promising candidate for preclinical optimization and future clinical evaluation in IPF.

## Data Availability

Source data for all figures are provided and available in this paper.

## Supplementary Materials

The following supporting information can be downloaded at https://www.mdpi.com/article/10.3390/medicina61071157/s1: Table S1: Primer sequences.

## References

[1] Moss B.J., Ryter S.W., Rosas I.O. (2022). Pathogenic Mechanisms Underlying Idiopathic Pulmonary Fibrosis. Annu. Rev. Pathol..

[2] Richeldi L., Collard H.R., Jones M.G. (2017). Idiopathic pulmonary fibrosis. Lancet.

[3] Spagnolo P., Kropski J.A., Jones M.G., Lee J.S., Rossi G., Karampitsakos T., Maher T.M., Tzouvelekis A., Ryerson C.J. (2021). Idiopathic pulmonary fibrosis: Disease mechanisms and drug development. Pharmacol. Ther..

[4] Raghu G., Remy-Jardin M., Richeldi L., Thomson C.C., Inoue Y., Johkoh T., Kreuter M., Lynch D.A., Maher T.M., Martinez F.J. (2022). Idiopathic Pulmonary Fibrosis (an Update) and Progressive Pulmonary Fibrosis in Adults: An Official ATS/ERS/JRS/ALAT Clinical Practice Guideline. Am. J. Respir. Crit. Care Med..

[5] Finnerty J.P., Ponnuswamy A., Dutta P., Abdelaziz A., Kamil H. (2021). Efficacy of antifibrotic drugs, nintedanib and pirfenidone, in treatment of progressive pulmonary fibrosis in both idiopathic pulmonary fibrosis (IPF) and non-IPF: A systematic review and meta-analysis. BMC Pulm. Med..

[6] Glass D.S., Grossfeld D., Renna H.A., Agarwala P., Spiegler P., DeLeon J., Reiss A.B. (2022). Idiopathic pulmonary fibrosis: Current and future treatment. Clin. Respir. J..

[7] Bonella F., Spagnolo P., Ryerson C. (2023). Current and Future Treatment Landscape for Idiopathic Pulmonary Fibrosis. Drugs.

[8] Amati F., Stainer A., Polelli V., Mantero M., Gramegna A., Blasi F., Aliberti S. (2023). Efficacy of Pirfenidone and Nintedanib in Interstitial Lung Diseases Other than Idiopathic Pulmonary Fibrosis: A Systematic Review. Int. J. Mol. Sci..

[9] Lee J.-W., Chun W., Lee H.J., Min J.-H., Kim S.-M., Seo J.-Y., Ahn K.-S., Oh S.-R. (2021). The Role of Macrophages in the Development of Acute and Chronic Inflammatory Lung Diseases. Cells.

[10] Richards C.D. (2017). Innate Immune Cytokines, Fibroblast Phenotypes, and Regulation of Extracellular Matrix in Lung. J. Interferon Cytokine Res..

[11] Adams T.S., Schupp J.C., Poli S., Ayaub E.A., Neumark N., Ahangari F., Chu S.G., Raby B.A., DeIuliis G., Januszyk M. (2020). Single-cell RNA-seq reveals ectopic and aberrant lung-resident cell populations in idiopathic pulmonary fibrosis. Sci. Adv..

[12] CPerrot Y., Karampitsakos T., Herazo-Maya J.D. (2023). Monocytes and macrophages: Emerging mechanisms and novel therapeutic targets in pulmonary fibrosis. Am. J. Physiol. Cell Physiol..

[13] Mantovani A., Sica A., Sozzani S., Allavena P., Vecchi A., Locati M. (2004). The chemokine system in diverse forms of macrophage activation and polarization. Trends Immunol..

[14] Chana K.K., Fenwick P.S., Nicholson A.G., Barnes P.J., Donnelly L.E. (2014). Identification of a distinct glucocorticosteroid-insensitive pulmonary macrophage phenotype in patients with chronic obstructive pulmonary disease. J. Allergy Clin. Immunol..

[15] Vogel D.Y., Glim J.E., Stavenuiter A.W., Breur M., Heijnen P., Amor S., Dijkstra C.D., Beelen R.H. (2014). Human macrophage polarization in vitro: Maturation and activation methods compared. Immunobiology.

[16] Heukels P., Moor C.C., von der Thüsen J.H., Wijsenbeek M.S., Kool M. (2019). Inflammation and immunity in IPF pathogenesis and treatment. Respir. Med..

[17] Koudstaal T., Wijsenbeek M.S. (2023). Idiopathic pulmonary fibrosis. La Presse Médicale.

[18] Arora S., Dev K., Agarwal B., Das P., Syed M.A. (2018). Macrophages: Their role, activation and polarization in pulmonary diseases. Immunobiology.

[19] Cheng P., Li S., Chen H. (2021). Macrophages in Lung Injury, Repair, and Fibrosis. Cells.

[20] Kishore A., Petrek M. (2021). Roles of Macrophage Polarization and Macrophage-Derived miRNAs in Pulmonary Fibrosis. Front. Immunol..

[21] Pokhreal D., Crestani B., Helou D.G. (2023). Macrophage Implication in IPF: Updates on Immune, Epigenetic, and Metabolic Pathways. Cells.

[22] Peng L., Wen L., Shi Q.-F., Gao F., Huang B., Meng J., Hu C.-P., Wang C.-M. (2020). Scutellarin ameliorates pulmonary fibrosis through inhibiting NF-κB/NLRP3-mediated epithelial–mesenchymal transition and inflammation. Cell Death Dis..

[23] Wu X., Wang Z., Shi J., Yu X., Li C., Liu J., Zhang F., Chen H., Zheng W. (2022). Macrophage polarization toward M1 phenotype through NF-κB signaling in patients with Behçet’s disease. Arthritis Res. Ther..

[24] Lawrence T. (2009). The nuclear factor NF-kappaB pathway in inflammation. Cold Spring Harb. Perspect. Biol..

[25] Sun S.-C. (2017). The non-canonical NF-κB pathway in immunity and inflammation. Nat. Rev. Immunol..

[26] Jaffar J., Glaspole I., Symons K., Westall G. (2021). Inhibition of NF-κB by ACT001 reduces fibroblast activity in idiopathic pulmonary fibrosis. Biomed. Pharmacother. = Biomed. Pharmacother..

[27] Ren L., Konger R.L. (2019). Evidence that peroxisome proliferator-activated receptor γ suppresses squamous carcinogenesis through anti-inflammatory signaling and regulation of the immune response. Mol. Carcinog..

[28] Liu Y., Wang J., Luo S., Zhan Y., Lu Q. (2020). The roles of PPARγ and its agonists in autoimmune diseases: A comprehensive review. J. Autoimmun..

[29] Ju Z., Su M., Hong J., Kim E., Jung J.H. (2020). Anti-inflammatory effects of an optimized PPAR-γ agonist via NF-κB pathway inhibition. Bioorg. Chem..

[30] Yang J., Zhou Y., Guan Y. (2012). PPARγ as a therapeutic target in diabetic nephropathy and other renal diseases. Curr. Opin. Nephrol. Hypertens..

[31] Imig J.D., Merk D., Proschak E. (2021). Multi-Target Drugs for Kidney Diseases. Kidney360.

[32] Cheng H.S., Tan W.R., Low Z.S., Marvalim C., Lee J.Y.H., Tan N.S. (2019). Exploration and Development of PPAR Modulators in Health and Disease: An Update of Clinical Evidence. Int. J. Mol. Sci..

[33] Nabi S., Bhandari U., Haque S.E. (2022). Saroglitazar ameliorates monosodium glutamate-induced obesity and associated inflammation in Wistar rats: Plausible role of NLRP3 inflammasome and NF-κB. Iran. J. Basic Med. Sci..

[34] Gawrieh S., Noureddin M., Loo N., Mohseni R., Awasty V., Cusi K., Kowdley K.V., Lai M., Schiff E., Parmar D. (2021). Saroglitazar, a PPAR-α/γ Agonist, for Treatment of NAFLD: A Randomized Controlled Double-Blind Phase 2 Trial. Hepatology.

[35] Roy A., Tewari B., Giri S., Goenka M. (2023). Saroglitazar in Non-alcoholic Fatty Liver Disease from Bench to Bedside: A Comprehensive Review and Sub-group Meta-Analysis. Cureus.

[36] Joshi S.R. (2015). Saroglitazar for the treatment of dyslipidemia in diabetic patients. Expert Opin. Pharmacother..

[37] Bandyopadhyay S., Samajdar S.S., Das S. (2023). Effects of saroglitazar in the treatment of non-alcoholic fatty liver disease or non-alcoholic steatohepatitis: A systematic review and meta-analysis. Clin. Res. Hepatol. Gastroenterol..

[38] Lakatos H.F., Thatcher T.H., Kottmann R.M., Garcia T.M., Phipps R.P., Sime P.J. (2007). The Role of PPARs in Lung Fibrosis. PPAR Res..

