# Supplementary material for: Saroglitazar Ameliorates Pulmonary Fibrosis Progression in Mice by Suppressing NF-κB Activation and Attenuating Macrophage M1 Polarization
Source: Medicina (Kaunas). 2025 Jun 26;61(7):1157. doi: 10.3390/medicina61071157 (PMC12299589; doi:10.3390/medicina61071157)
Supplement: Supplementary file 1 [file medicina-61-01157-s001.zip › medicina-3624785-supplementary.pdf]

medicina

Supplementary Table S1

Primer sequences

Gene name	Sequence
Il6	Forward 5'-3' TAGTCCTTCCTACCCCAATTTCC
	Reward 5'-3' TTGGTCCTTAGCCACTCCTTC
Il1b	Forward 5'-3' GCAACTGTTCCTGAACTCAACT
	Reward 5'-3' ATCTTTTGGGGTCCGTCAACT
Tnf	Forward 5'-3' CCCTCACACTCAGATCATCTTCT
	Reward 5'-3' GCTACGACGTGGGCTACAG
nlrp3	Forward 5'-3' ATTACCCGCCCGAGAAAGG
	Reward 5'-3' TCGCAGCAAAGATCCACACAG
Pycard	Forward 5'-3' CTTGTCAGGGGATGAACTCAAAA
	Reward 5'-3' GCCATACGACTCCAGATAGTAGC
Nfkb	Forward 5'-3' AACAGAGAGGATTTCGTTTCCG
	Reward 5'-3' TTTGACCTGAGGGTAAGACTTCT
Actb	Forward 5'-3' GGCTGTATTCCCCTCCATCG
	Reward 5'-3' CCAGTTGGTAACAATGCCATGT

 https://doi.org/10.3390/medicina61071157
